# DNA barcoding reveals both known and novel taxa in the Albitarsis Group (*Anopheles*: *Nyssorhynchus*) of Neotropical malaria vectors

**DOI:** 10.1186/1756-3305-5-44

**Published:** 2012-02-21

**Authors:** Freddy Ruiz-Lopez, Richard C Wilkerson, Jan E Conn, Sascha N McKeon, David M Levin, Martha L Quiñones, Marinete M Póvoa, Yvonne-Marie Linton

**Affiliations:** 1 Entomology Branch, Walter Reed Army Institute of Research, 503 Robert Grant Avenue, Silver Spring, Maryland 20910, USA; 2 Griffin Laboratory, Wadsworth Center, New York State Department of Health, Albany, New York, USA; 3 Department of Biomedical Sciences, School of Public Health, State University of New York, Albany, New York, USA; 4 Facultad de Medicina, Universidad Nacional de Colombia, Bogotá D.C., Colombia; 5 Instituto Evandro Chagas, Ananindeua, Pará, Brazil; 6 Department of Entomology, Natural History Museum, London, UK

**Keywords:** Albitarsis Group, barcoding, *COI*, new species, *An. albitarsis *G, *An. albitarsis *H, *An. albitarsis *I

## Abstract

**Background:**

Mosquitoes belonging to the Albitarsis Group (*Anopheles*: *Nyssorhynchus*) are of importance as malaria vectors across the Neotropics. The Group currently comprises six known species, and recent studies have indicated further hidden biodiversity within the Group. DNA barcoding has been proposed as a highly useful tool for species recognition, although its discriminatory utility has not been verified in closely related taxa across a wide geographic distribution.

**Methods:**

DNA barcodes (658 bp of the mtDNA *Cytochrome c Oxidase *- *COI*) were generated for 565 *An. albitarsis *s.l. collected in Argentina, Brazil, Colombia, Paraguay, Trinidad and Venezuela over the past twenty years, including specimens from type series and type localities. Here we test the utility of currently advocated barcoding methodologies, including the Kimura-two-parameter distance model (K2P) and Neighbor-joining analysis (NJ), for determining species delineation within mosquitoes of the Neotropical Albitarsis Group of malaria vectors (*Anopheles*: *Nyssorhynchus*), and compare results with Bayesian analysis.

**Results:**

Species delineation through barcoding analysis and Bayesian phylogenetic analysis, fully concur. Analysis of 565 sequences (302 unique haplotypes) resolved nine NJ tree clusters, with less than 2% intra-node variation. Mean intra-specific variation (K2P) was 0.009 (range 0.002 - 0.014), whereas mean inter-specific divergence were several-fold higher at 0.041 (0.020 - 0.056), supporting the reported "barcoding gap". These results show full support for separate species status of the six known species in the Albitarsis Group (*An. albitarsis *s.s., *An. albitarsis *F, *An. deaneorum*, *An. janconnae*, *An. marajoara *and *An. oryzalimnetes*), and also support species level status for two previously detected lineages - *An. albitarsis *G &*An. albitarsis *I (designated herein). In addition, we highlight the presence of a unique mitochondrial lineage close to *An. deaneorum *and *An. marajoara *(*An. albitarsis *H) from Rondônia and Mato Grosso in southwestern Brazil. Further integrated studies are required to confirm the status of this lineage.

**Conclusions:**

DNA barcoding provides a reliable means of identifying both known and undiscovered biodiversity within the closely related taxa of the Albitarsis Group. We advocate its usage in future studies to elucidate the vector competence and respective distributions of all eight species in the Albitarsis Group and the novel mitochondrial lineage (*An. albitarsis *H) recovered in this study.

## Background

The need to understand the systematics of the Neotropical Albitarsis Group (*Anopheles *subgenus *Nyssorhynchus*) [1] is primarily driven by the operational requirement to reliably distinguish which component taxa are involved in malaria transmission. The group is of great epidemiological importance as three of the five formally described species are proven regional malaria vectors in Brazil: *An. deaneorum *Rosa-Freitas [2-4], *An. janconnae *Wilkerson and Sallum (= *An. albitarsis *E) [5] and *An. marajoara *Galvão and Damasceno [6-8]. The vector status of *An. oryzalimnetes *Wilkerson and Motoki (= *An. albitarsis *B), *An. albitarsis *Lynch-Arribálzaga and the informally named species *An. albitarsis *"F" [9], are unknown. The group, along with *An. braziliensis*, is easily recognized in the adult stage by a pair of white scale stripes on sternum I [10]. The taxonomy of this group is historically complex and a comprehensive review is given in Motoki *et al. *[11].

Recent studies using *COI *and *white *gene sequences [12], in addition to the complete mitochondrion DNA [13] for five species belonging to Albitarsis Group, suggested that *An. marajoara *from Manaus (Brazil) should be regarded as a separate taxon in the Group (= *An. albitarsis *G) [12,13]. In the past year, two further lineages have been proposed suggesting that species discovery in the Albitarsis Group is far from complete. One, closely related to *An. janconnae*, was detected in the Caribbean region of Colombia based on *COI*, *white *gene and second internal transcribed spaces (ITS2) sequences [14]. The other, closely related to *An. deaneorum*, was found in Acrelândia, Acre, Brazil, using *white *and *NADH dehydrogenase subunit 6 *(*ND6*) gene sequences [15]. Neither of these proposed lineages was named.

Sequence data exist for mitochondrial *COI *[12,13,16,17], *NADH dehydrogenase subunit 4 *(*ND4*) [17], *ND6 *[17] and whole mitochondrial genomes [13], and the nuclear *white *[12,14], ITS2 [9,14,18] and Dominant receptor (D2) [18] regions. However, a single marker is still to be identified, which can to separate all known species and recognized lineages in the Albitarsis Group to ensure accurate species identification for studies on vector competence, for accurate distribution mapping, and to facilitate vector control efforts. Early attempts to use the ITS2 as a species diagnostic marker in the group [19] were hampered by the inadvertent inclusion of undiscovered taxa [9,13,14] and extensive intragenomic variation, which rendered the proposed ITS2-PCR assay unusable [20].

Mitochondrial genes are considered better markers than nuclear genes because of their abundance (1000's copies per cell), lack of introns, limited exposure to recombination, and haploid mode of inheritance [21]. DNA barcoding - which relies on the genetic variation within a standardized region of the *COI *gene - has been promoted as a reliable method for the identification of species in a variety of both invertebrate and vertebrate taxa [22]. Krzywinski *et al. *[13] sequenced whole mitochondrial genomes of six confirmed and putative taxa in the Albitarsis Group and showed that the 5' half of the *COI *"barcode region" is clearly more variable than its 3' half. *COI *barcoding studies of the mosquito fauna of Canada [23] and India [24], and within the genus *Anopheles *[24-27] have shown exceptional promise for species-level determinations. However, there has been no rigorous testing of the utility of the barcoding region in large populations of geographically widespread and closely related species such as those in the Albitarsis Group.

Herein we investigate the utility of the standard DNA *COI *barcode region for species identification using both the standard "simple" barcode methodology [22], NJ [28] and K2P model [29] to differentiate known and unknown species from 565 members of the Albitarsis Group, collected across a wide geographic range in South America in the last 20 years. For comparison, a subset of the full dataset is further investigated in a phylogenetic Bayesian framework [30].

## Methods

### Specimens and data access

Full specimen records (collection locality, coordinates, specimen identifiers, location of voucher specimens etc.) and all genetic data (edited chromatograms, consensus *COI *sequence files and corresponding GenBank numbers) are publicly accessible under the project code MBIK (Albitarsis Group Barcoding) on the BOLD website (http://www.boldsystems.org), as part of the Mosquito Barcoding Initiative (MBI). Collection data and distribution maps are also publicly available through MosquitoMap (http://www.mosquitomap.org). Specimens utilized in the molecular study were all morphologically verified as *An. albitarsis *s.l., using the original descriptions, and available keys [1,31] and include topotypic material for *An. albitarsis *s.s., *An. deaneorum*, *An. marajoara *and *An. oryzalimnetes*, and type series material of *An. janconnae*. Voucher specimens and/or their DNA extracts for the majority of specimens used in this study are stored at -80°C in the archive collections of Walter Reed Biosystematics Unit, Smithsonian Institution, Museum Support Center, Suitland, Maryland, USA, or in the Culicid DNA Collection of the Molecular Systematics Laboratory, Natural History Museum, London.

### Sequence generation

DNA barcodes (658 bp, excluding primers) were generated for 565 specimens collected across South America using the published high-throughput DNA extraction and PCR amplification protocols [27] using the LCO1490 & HCO2198 primers of Folmer *et al. *[32]. Sequencing reactions were carried out in both directions with the Big Dye^® ^Terminator Kit on an ABI 3730 automated sequencer (PE Applied Biosystems). Sequences were edited in Sequencher™ v4.8 (Genes Codes Corporation, Ann Arbor, MI), and translated to amino acids in MacClade v.4.06 [33]. Similarities with publicly available sequences were assessed using BLAST (Basic Local Alignment Search Tool), available at http://blast.ncbi.nlm.nih.gov/Blast.cgi, and comparisons with unpublished barcode records checked through the IDS (Identification System) of the Barcode of Life database (BOLD, available at http://www.barcodinglife.org).

### Data analysis

To test the resolution of customarily advocated barcoding methodologies [22], 302 unique haplotypes (n = 565) *COI *barcodes were first imported into PAUP* v.4.0 [34]. Following construction of a pairwise distance matrix using K2P [29], a bootstrapped [35] NJ tree [28] was generated using 1,000 replicates to produce an unrooted consensus tree. Sequence divergence was calculated in MEGA v.4.0 [36] using the K2P model. *Anopheles braziliensis *Chagas [GenBank: DQ076238] and *An. darlingi *Root [GenBank: DQ076236] were used as outgroup taxa to the Albitarsis Group, following previously published studies [14,16].

A Bayesian phylogenetic analysis [30] was carried out using five specimens from each distinctive *COI *lineage found by NJ-K2P analysis (including respective type localities where possible). These sequences were added to the publicly available *COI *sequences of [14,16] (trimmed to 658 bp). Recently published *COI *data [12,13] overlapped those already included in our dataset and were therefore not included in the analysis. MrBayes [30] was performed online at: http://cbsuapps.tc.cornell.edu/mrbayes.aspx. The Bayesian analysis was run for 10 million generations with two parallel searches using three heated and one cold Markov chain, with the first 5 million generations discarded as burn-in. The best-fit model HKY + I + G was selected by the AIC criterion in MrModeltest 2.3 [37]. The trees generated were edited in Figtree v1.2.1 [38].

## Results

### Sequence statistics

MtDNA *COI *sequences (658 bp) were generated for 565 *An. albitarsis *s.l. collected across South America in the past twenty years: Argentina (n = 38), Brazil (n = 448), Colombia (n = 21), Paraguay (n = 23), Trinidad (n = 19) and Venezuela (n = 16). Among the 565 sequences were 302 unique haplotypes. Average nucleotide composition percentages for all 565 Albitarsis Group sequences were 38.5% (T), 15.8% (C), 29.4% (A) and 16.2% (G) (Table 1). Alignments were unambiguous: amino acid translations showed no stop codons, indicating that all sequences represented functional protein coding genes, not pseudogenes. The amino acid reading frame starts at the second base of the primer-edited sequences. The 302 unique nucleotide haplotypes, translated to 14 unique AA sequences (data not shown). All species in the Albitarsis Group were represented by the most common AA sequence, therefore AA derived phylogenies are not informative for species differentiation within this group.

**Table 1:** Mean pairwise nucleotide frequencies of *COI *barcode sequences for individuals belonging to Albitarsis Group.

	Identical pairs	No. of transitions (TS %)	No. of transversions (TV %)	T	C	A	G	Total bases
**Average**	634	19 (2.8)	5 (0.8)	38.5	15.8	29.4	16.2	658
**1st**	217	2 (0.9)	0	27	14.8	27.9	30.1	219
**2nd**	219	0	0	43	26.9	13.7	16.4	219
**3rd**	199	17 (8)	5 (2.3)	45	5.9	46.5	2.2	220

### Determination of barcode clusters

**Figure 1:**
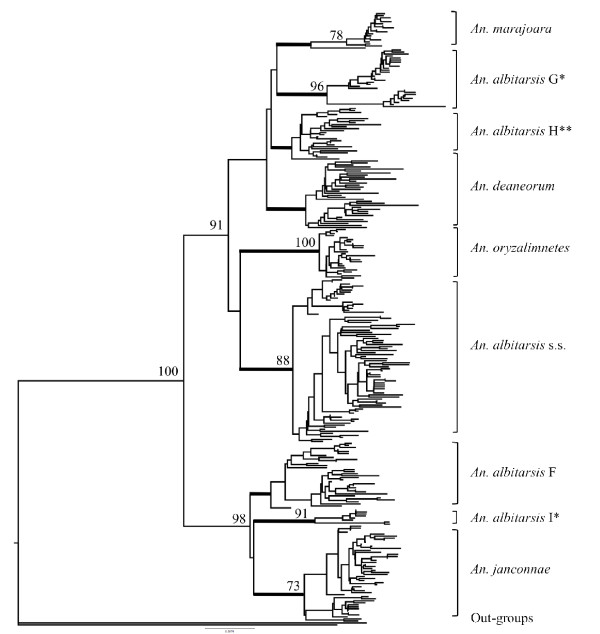
**Bootstrapped NJ tree, based on 1000 replicates of K2P data matrices**. Bootstrapped NJ tree of 302 unique *COI *haplotypes generated from 565 individuals belonging to Albitarsis Group, based on K2P distance algorithm. Bootstrap values less than 70% are not shown. *Highlights taxa regarded as species in this study. **Novel lineage. Outgroup taxa include *An. darlingi *[GenBank: DQ076236] and *An. braziliensis *[GenBank: DQ076238]. The bold lines indicate the nine phylogroups considered here.

Following NJ-K2P analysis, nine distinct clusters were recovered (Figure 1), six of which corresponded to the previously determined species (*An. albitarsis *s.s. (n = 109), *An. albitarsis *F (n = 44), *An. deaneorum *(n = 36), *An. marajoara *(n = 70)*, An. janconnae *(n = 96), and *An. oryzalimnetes *(n = 51), with a further three clusters corresponding to the previously described lineages *An. albitarsis *G (n = 105) [12,13] and sp. nr. *An. janconnae *[14] (herein called *An. albitarsis *I) (n = 12), and a newly detected lineage designated herein as *An. albitarsis *H (n = 42). *Anopheles albitarsis *s.s., *An. janconnae, An. marajoara *and *An. oryzalimnetes *were supported by high bootstrap values (BSV) (88%, 73%, 78% and 100%, respectively), as was *An. albitarsis *G (BSV 96%) and the additional lineage *An. albitarsis *I (BSV 92%). However, *An. deaneorum*, *An. albitarsis *F and the new lineage *An. albitarsis *H were poorly supported with BSV's of < 70%.

**Figure 2:**
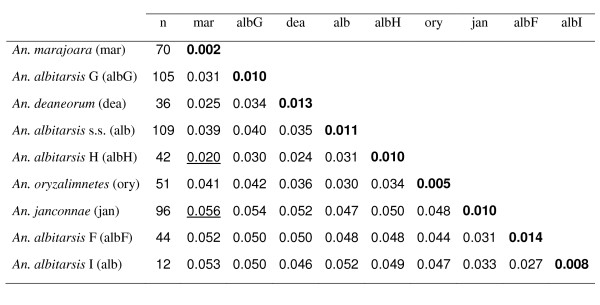
**Mean inter- and intra- specific of K2P distances in nine species clusters**. The species clusters were defined using the NJ-K2P distance method commonly utilized in barcoding. Numbers in boldface indicate intra-specific genetic divergence. Numbers underlined indicate the lowest (*An. marajoara *with *An. albitarsis *H) and highest (*An. janconnae *with *An. marajoara*) values between the species and lineages. Specimen numbers per species are indicated in parentheses.

**Figure 3:**
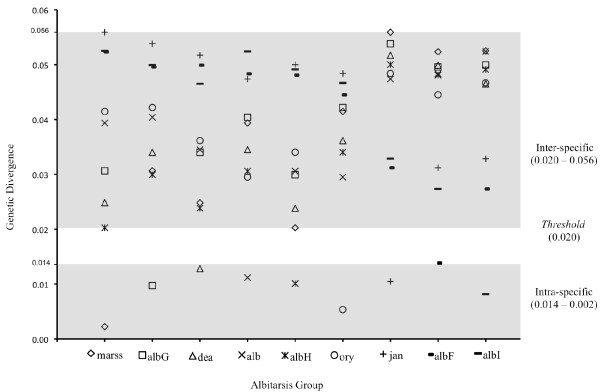
**Plot of K2P distance matrices of the nine clusters determined using NJ-K2P distances**. Y-axis: genetic divergence and X-axis clusters. **mar**: *An. marajoara*; **albG**: *An. albitarsis *G; **dea**: *An. deaneorum*; **alb**: *An. albitarsis *s.s.; **albH**: *An. albitarsis *H; **ory**: *An. oryzalimnetes*; **jan**: *An. janconnae*; **albF**: *An. albitarsis *F; **albI**: *An. albitarsis *I.

Genetic divergence was estimated based on the nine clusters determined with NJ-K2P analysis (Figure 2, Figure 3). The overall mean distance (K2P) was 0.037. The average intra-specific genetic divergence was 0.009 (0.002 - 0.014) and the average inter-specific divergence was 0.04 (0.020 to 0.056). The most genetically divergent clusters were *An. marajoara *with *An. janconnae *(0.056) and *An. janconnae *with *An. albitarsis *G (0.054); while the most similar pairs were *An. albitarsis *H - *An. marajoara *(0.020) and *An. albitarsis *H -* An. deaneorum *(0.024) (Figure 2, Figure 3). *Anopheles albitarsis *I, with a mean genetic divergence of 0.045 (range 0.027 - 0.053), showed consistently higher genetic divergence with respect to all other taxa in the Albitarsis Group (Figure 2, Figure 3).

### Bayesian Analysis

**Figure 4:**
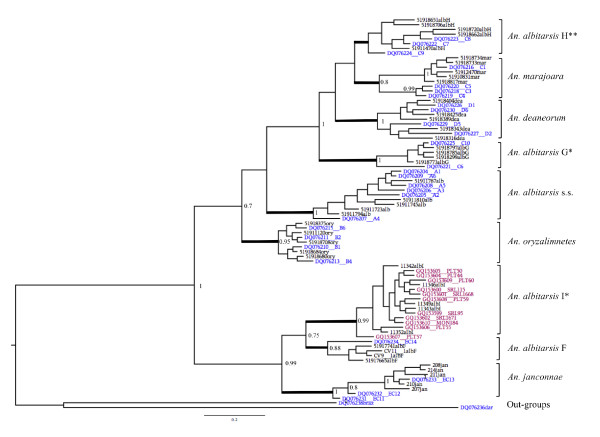
**MrBayes tree generated using GenBank sequences and topotypic specimens and lineages found with NJ-K2P distances**. Bold lines indicate the nine phylogroups considered here: **albH**: *An. albitarsis *H; **mar**: *An. marajoara*; **dea**: *An. deaneorum*; **albG**: *An. albitarsis *G; **alb**: *An. albitarsis *s.s.; **ory**: *An. oryzalimnetes*; **albI**: *An. albitarsis *I; **albF**: *An. albitarsis *F; **jan**: *An. janconnae*. Outgroups: *An. darlingi *and *An. braziliensis*. P values less than 70% are not shown. *Newly determined species. **Novel lineage. *COI *sequences from previously published works are indicated in blue [16] and red [14].

**Figure 5:**
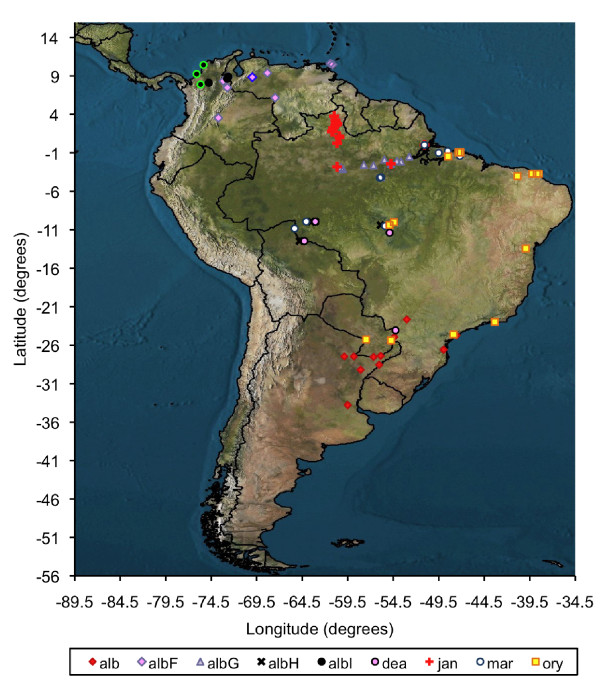
**Map of South America showing the verified distributions of members of the Albitarsis Group based on *COI *DNA sequences**. Locality data for 565 *An. albitarsis *s.l. specimens were plotted alongside those GenBank sequences whose identity has been molecularly verified within [14,16]. **alb**: *An. albitarsis *s.s.; **albF**: *An. albitarsis *F; **albG**: *An. albitarsis *G; **albH**: *An. albitarsis *H; **albI: ***An. albitarsis *I; **dea**: *An. deaneorum*; **jan**: *An. janconnae*; **mar**: *An. marajoara*; **ory**: *An. oryzalimnetes*. Localities determined from GenBank *COI *sequences are indicated as follow: *An. albitarsis *F [GenBank: DQ076234] [16], fuchsia diamond with heavy blue edging, and *An. albitarsis *I [GenBank: GQ153597-610] [14] black circles with bright green edging.

Bayesian analysis supported all formally described species and previously indicated lineages detected with NJ-K2P (posterior probability P = 0.8 - 1), however, the newly recognized lineage, *An. albitarsis *H, is unresolved (P = 0.4, not shown) (Figure 4). The monophyly of the Albitarsis Group is confirmed and four species groups are recognized as follow: Group 1, *An. janconnae*, *An. albitarsis *F and *An. albitarsis *I; Group 2, *An. albitarsis *G, *An. albitarsis *H, *An. deaneorum *and *An. marajoara*; Group 3, *An. albitarsis *s.s.; Group 4, *An. oryzalimnetes*. Groups 2, 3 and 4 are more closely related, and they are paraphyletic with respect to Group 1. The lineage proposed by Gutierrez *et al. *[14] from the Colombian Caribbean coast showed strong correlation (P = 0.99) with *An. albitarsis *I. Lehr *et al. *[16] undetermined *COI *sequences grouped here with *An. albitarsis *G and *An. albitarsis *H (Figure 4) (see Figure 5), [16].

## Discussion

The analysis of *COI *sequences from members of the Albitarsis Group using NJ-K2P distances revealed nine distinct clusters: six of these correspond to the formerly defined species *An. albitarsis *s.s., *An. deaneorum*, *An. janconnae*, *An. marajoara*, *An. oryzalimnetes *and *An. albitarsis *F, and another to *An. albitarsis *G [13], which we believe to comprise a separate species in the Albitarsis Group along with "sp. nr. *An. janconnae" *[14], named *An. albitarsis *I. The remaining cluster, which we believe to represent a separate lineage, is named *An. albitarsis *H. Currently available synonyms for the Albitarsis Group as discussed in Motoki *et al. *[11] (*An. albitarsis *(*Anopheles albitarsis *var. *limai *Galvão and Lane, *Anopheles albitarsis imperfectus *Corrêa and Ramos)), *An. marajoara *(*An. albitarsis domesticus *Galvão and Damasceno) and the status *An. allopha *as a *nomen dubium *[1] are in our opinion sustained, with none referring to the newly recognized species/lineages presented here.

The monophyly of the Albitarsis Group is strongly supported with a high P value (P = 1), which echoes earlier studies [14-17]. BLAST searches of *COI *sequences revealed 99-100% homology with sequences reported for *An. albitarsis *s.s. [GenBank: DQ076204-DQ076208], *An. deaneorum *[GenBank: DQ076226, DQ076230], *An. janconnae *[GenBank: DQ076233], *An. marajoara *[GenBank: DQ076216] and *An. oryzalimnetes *[GenBank: DQ076210-DQ076215] [16]. This further reinforces our confidence in the identities of these taxa. The respective distributions and taxonomic positions of the informally designated taxa (*An. albitarsis *F, G, and I) and new lineage *An. albitarsis *H within the Albitaris Group are discussed in relation to earlier works below.

### *Anopheles albitarsis *F

*Anopheles albitarsis *F was originally proposed as a putative new species in the Albitarsis Group based on ITS2 and *white *gene sequences [9] from specimens collected in Vichada, Colombia. One of the individuals assumed by Lehr *et al. *[16] to be *An. albitarsis *E (= *An. janconnae*), was unresolved in their Bayesian phylogeny (sample C14, Portuguesa, Venezuela [GenBank: DQ076234]), but this was confirmed as *An. albitarsis *F in our Bayesian analysis (Figure 4). Correlation of our data with these earlier studies confirms the wider distribution of the species outside of Colombia. As well as Vichada, Colombia [9] and Portuguesa, Venezuela [16], we further report it from the states of Cojedes and Zulia in Venezuela and in St George East and St Andrew/St David in Trinidad (Table 2, Figure 5).

**Table 2:** List of species found by country and state, belonging to Albitarsis Group.

Country	N =	States	Species
**Argentina**	38	Buenos Aires, Corrientes, Misiones	*An. albitarsis *s.s.
**Brazil**	57	Paraná, Santa Catarina, São Paulo	*An. albitarsis *s.s.
	105	Amazonas, Bahia, Pará	*An. albitarsis *G
	42	Mato Grosso, Rondônia	*An. albitarsis *H
	36	Mato Grosso, Rondônia, Paraná	*An. deaneorum*
	96	Amapá, Roraima, Pará	*An. janconnae*
	70	Amapá, Mato Grosso, Pará, Rondônia	*An. marajoara*
	42	Espirito Santo, Bahia, Ceará, Mato Grosso Pará, Rio de Janeiro, Rondônia, São Paulo	*An. oryzalimnetes*
**Colombia**	11	Antioquia, Norte de Santander	*An. albitarsis *I
	10	Meta, Norte de Santander, Vichada	*An. albitarsis *F
**Paraguay**	14	Alto Paraná	*An. albitarsis *s.s.
	9	Alto Paraná	*An. oryzalimnetes*
**Trinidad**	19	St. George East, St. Andrew/St.David	*An. albitarsis *F
**Venezuela**	15	Cojedes, Zulia	*An. albitarsis *F
	1	Zulia	*An. albitarsis *I

**Total**	565		

The coordinates and collection information are available at www.mosquitomap.org and on the BOLD website (www.boldsystems.org) under the Mosquitoes of the World Project MBIK. GenBank accessions are: *An. albitarsis *s.s. [GenBank: JQ615201-JQ615309], *An. deaneorum *[GenBank: JQ615310-JQ615345], *An. janconnae *[GenBank: JQ615346-JQ615441], *An. marajoara *[GenBank: JQ615442-JQ615511], *An. oryzalimnetes *[GenBank: JQ615512-JQ615562], *An. albitarsis *F [GenBank: JQ614998-JQ615041], *An. albitarsis *G [GenBank: JQ615042-JQ615146], *An. albitarsis *H [GenBank: JQ615147-JQ615188] and *An. albitarsis *I [GenBank: JQ615189-JQ615200].

Morphologically, *An. albitarsis *F is similar to the proven malaria vector *An. marajoara *[9], thus the wider distribution of this cryptic taxon in Venezuela and Trinidad is of epidemiological significance. Based on morphology and RAPD profiling [18], *An. marajoara *was believed to be the only member of the Albitarsis Group present in western Venezuela [8], where it is a proven secondary vector of *Plasmodium vivax *210 [39]. However, these studies predate the discovery of *An. albitarsis *F and it is not known if the species-diagnostic RAPD assay (developed for the four known members of the Albitarsis Group at that time) [40] could distinguish *An. marajoara *from *An. albitarsis *F. Given the data presented herein, it seems likely that "*An. marajoara" *reported in Venezuela [8] corresponds to *An. albitarsis *F. It is also probable that "population C" from Venezuela and Colombia [41,42] identified on the basis of chromosomal analysis, may also be *An. albitarsis *F. Further sampling is needed to assess the true identity of *An. albitarsis *s.l. in Venezuela and assess whether *An. albitarsis *F is the only member of the group present there, or whether *An. marajoara *and *An. albitarsis *F are sympatric in that country. This is also true for the population of *An. marajoara *in Trinidad, which is regarded as a secondary vector in the region [43]. An ecological study stated that *An. marajoara *was identified from the island based on RAPD profiles and ITS2 sequences [43], yet our data clearly show all samples tested from Trinidad to be *An. albitarsis *F. Concurrent species determination, distribution mapping and vector incrimination studies are needed to assess the true impact of *An. marajoara *and *An. albitarsis *F on malaria transmission across their range.

### *Anopheles albitarsis *G

Mean *COI *genetic intra-specific distance within *An. albitarsis *G was 0.010, whereas mean inter-specific divergence within the Albitarsis Group were four-fold higher (0.041) (Figure 2, Figure 3), and well above accepted species delimitation divergence thresholds routinely employed in DNA barcoding [22-27]. Based on consistent differences in DNA sequence in one individual (C10 [GenBank: DQ076225]) from four different DNA markers [40], Wilkerson *et al*. first suggested the presence of hidden genetic diversity within *An. marajoara *from Manaus, Brazil. Bayesian analysis of the entire *COI *gene showed that sample C10 grouped closest with *An. deaneorum*, but with low support (P = 0.69) [16]. Because of this, these authors questioned the validity of *An. deaneorum *arguing: "if *An. deaneorum *is a separate species from *An. marajoara*, then *An. marajoara *may consist of two or more species in Amazonian Brazil". The presence of *An. albitarsis *G as a cryptic species in the Albitarsis Group was later confirmed in further DNA studies [12,13]. BLAST of our *An. albitarsis *G sequences returned 100% homology with GenBank: DQ076225 (C10, from Manaus, Brazil, as *An. marajoara*) and GenBank: DQ076217 (C2, from Itaituba, Brazil, as *An. marajoara*) [16]. GenBank: DQ076221 (Itaituba, Brazil) [16] still shows deviation in the Bayesian phylogeny. However, at this time we are unable to assess whether this reflects further hidden genetic diversity, whether these differences are due to poor sequence quality in the first instance, or are indeed real and reflective of geographic distance as all other specimens sequenced are from Manaus, Brazil (Figure 4). Further sampling from Itaituba and other localities is needed.

### *Anopheles albitarsis *H

This novel mitochondrial lineage was detected in 42 specimens collected in Rondônia and Mato Grosso, Brazil. *COI *sequences showed highest similarity with *An. marajoara *and *An. deaneorum *(0.020 and 0.024, respectively), both values being significantly higher than its intra-specific divergence (0.010) (Figure 2, Figure 3). BLAST of our sequences revealed 99% homology with GenBank: DQ076222 from Matupá and GenBank: DQ076223 from Peixoto de Azevedo (both Mato Grosso, Brazil) and GenBank: DQ076224 from Ariquemes, Rondônia, Brazil (as *An. marajoara *samples C7, C8 and C9) [16]. These three samples were shown to form a cluster distinct from *An. marajoara*, which is further supported in our Bayesian analysis (Figure 4).

Based on sequence variation, the barcoding community generally accept species delineation if the intraspecific variation is less than 1%, while the inter-specific variation is at least 2% different from its nearest congener [22-27,44]. Average inter-specific divergences between known taxa in the Albitarsis Group is 0.036, however, inter-specific values between *An. deanorum*, *An. marajoara *and *Anopheles albitarsis *H are much lower, ranging from 0.020 - 0.025. Our *COI *data supports the suggestion of Lehr *et al. *[16], exposing this grouping as cryptic complex in its own right within the Albitarsis Group. The status of the mitochondrial lineage *An. albitarsis *H is far from resolved. Further detailed ecological, genetic and morphological studies are necessary before we can speculate whether this lineage is reflective of a new species within the Albitarsis Group.

The presence of a cryptic species near to *An. deaneorum *(using *ND6 *and *white *gene sequences [15]) and near to *An. marajoara *(by allozymes and mtDNA RFLPs [45]), were detected in populations of *An. albitarsis *s.l. from the same and neighbouring localities to where *An. albitarsis *H was detected in this study.

### *Anopheles albitarsis *I

Brochero *et al. *[9] first reported *An. albitarsis *F in Vichada, Colombia (east of the Cordillera Oriental) in sympatry with another taxon determined as *An. marajoara *based on morphological keys [10]. Microsatellite analysis of five populations of *An. marajoara *in Colombia revealed two incompletely isolated gene pools separated by the eastern Andean cordillera [46]. Using the entire *COI*, *white *gene and ITS2 sequences, Gutierrez *et al. *[14] reported a new lineage in the Albitarsis Group closely related to *An. janconnae *from northwestern Colombia. Sequences generated in our study share 99% homology with these *COI *sequences [GenBank: GQ153597-GQ153610] [14], and this cluster was highly supported in our Bayesian analysis (P = 0.99) (Figure 5). We clearly show that the lineage previously reported by Gutierrez *et al. *[14] as sp. nr. *An. janconnae *in the Caribbean region of Colombia and by Brochero *et al. *[46] as *An. marajoara*, is a separate species in the Albitarsis Group, which we call *An. albitarsis *I.

Despite the small sample size analysed, this species is clearly distinct from other members of the Albitarsis Group based on *COI *sequence data, with an intra-specific divergence of 0.008 and mean inter-specific divergence of 0.044 (0.027 with *An. albitarsis *F - 0.053 with *An. marajoara*) (Figure 2). That the sister taxon *An. albitarsis *F and *An. albitarsis *I were detected in sympatry in the geographically separate localities of Tibú, Norte de Santander, Colombia and Rio Socuavo, Zulia, Venezuela (Figure 5), provides further evidence to support their separate species status within the Albitarsis Group.

### Distribution of Albitarsis Group in South America based on *COI *sequences

Locality data for all molecularly confirmed Neotropical Albitarsis Group specimens used in this study (n = 565) and correlated *COI *data from previous studies [14,16] were plotted using the mapping methodology developed by Foley [47] (Figure 5). This figure shows the distributions of *An. albitarsis *F and *An. albitarsis *I to be restricted to northern South America (Colombia, Venezuela and Trinidad), whereas *An. albitarsis *s.s. is found in southern Brazil, northern Argentina and Paraguay. *Anopheles marajoara *is present in the central east and west region of Brazil and *An. deaneorum *is in southwestern Brazil. *Anopheles oryzalimnetes *has a wide distribution in Brazil, mainly in the central region (Figure 5). Three species in the Albitarsis Group and the new lineage *An. albitarsis *H appear endemic only to Brazil to date: *An. marajoara *(Amapá, Mato Grosso, Pará, Rondônia), *An. janconnae *is present in northern Brazil (Roraima and Pará states), *An. albitarsis *G in Amazonian Brazil (Amazonas and Pará states) and *An. albitarsis *H in Rondônia and Mato Grosso, Brazil. Some species and lineages were found to be sympatric: for example, *An. marajoara *with *An. oryzalimnetes *and *An. janconnae *in Pará, Brazil; *An. albitarsis *H, *An. deaneorum*, *An. marajaora *and *An. oryzalimnetes *in Mato Grosso, Brazil; *An. oryzalimnetes *and *An. albitarsis *in São Paulo, Brazil, amongst others (Figure 5).

Whilst this study significantly contributes to the verified distribution of members of the Albitarsis Group, it is important to note that samples were not available from Bolivia, Costa Rica, French Guiana, Guatemala, Guyana, Panama, Peru, Suriname and Uruguay, where species of the Group have previously been reported [48]. We advocate the utility of the *COI *barcoding region for the correct species identification of *An. albitarsis *s.l. specimens in these regions, allowing complete species level distribution maps to be constructed for all eight species and the new lineage recognised in this study.

## Conclusions

The results reported here using *COI *barcoding of specimens collected over 20 years from a wide geographic range in South America (Argentina, Brazil, Colombia, Paraguay, Trinidad and Venezuela), including topotypic specimens of the formally described taxa, helped resolve the taxonomic status and the distribution of the Albitarsis Group in South America. Contrary to intra-specific variation detected in previous studies using ITS2 [20], we consider the *COI *barcode region to be a robust marker of choice for species delimitation in the Albitarsis Group, not least because amplification of a short region (658 bp) yielded similar results to studies based on the entire mitochondria [13] or full *COI *gene [12,14,16]. We now consider the following species to comprise the Albitarsis Group: *An. albitarsis *s.s., *An. deaneorum*, *An. janconnae*, *An. marajoara*, *An. oryzalimnetes*, *An. albitarsis *F, *An. albitarsis *G and *An. albitarsis *I. In addition, we uncovered a new mitochondrial lineage, *An. albitarsis *H, which requires further sampling and sequencing of additional markers before its true species status can be resolved. This first comprehensive study of species in the Albitarsis Group across a wide geographic range in South America and provides a firm systematic basis for future studies that ideally should include ecology, biogeography, population genetics analysis, and vector incrimination of these species, particularly in malaria endemic regions where some of these new taxa may be acting as vectors.

## Competing interests

The authors declare that they have no competing interests.

## Authors' contributions

FRL, RCW & YML conceived the ideas; YML, RCW, MLQ & JEC obtained funding; FRL, SNM, JEC, MLQ & RCW undertook fieldwork and/or donated samples; FRL, YML & DML carried out the molecular laboratory work; FRL, YML, SNM & RCW carried out the data analysis and interpretation; FRL wrote the draft manuscript; YML, RCW, JEC, MMP, MLQ & SNM revised the draft manuscript; FRL, RCW & YML carried out the final revisions and submitted the manuscript. All authors read and approved the final version of the manuscript.
